# Mediator regulates non-coding RNA transcription at fission yeast centromeres

**DOI:** 10.1186/1756-8935-5-19

**Published:** 2012-11-21

**Authors:** Michael Thorsen, Heidi Hansen, Michela Venturi, Steen Holmberg, Genevieve Thon

**Affiliations:** 1 Department of Biology, University of Copenhagen, BioCenter, Ole Maaløes vej 5, 2200, Copenhagen, N, Denmark

**Keywords:** *S. pombe*, Chromatin, RNA Pol II, Mediator, Centromere, Chromosome segregation

## Abstract

**Background:**

In fission yeast, centromeric heterochromatin is necessary for the fidelity of chromosome segregation. Propagation of heterochromatin in dividing cells requires RNA interference (RNAi) and transcription of centromeric repeats by RNA polymerase II during the S phase of the cell cycle.

**Results:**

We found that the Med8-Med18-Med20 submodule of the Mediator complex is required for the transcriptional regulation of native centromeric *dh* and *dg* repeats and for the silencing of reporter genes inserted in centromeric heterochromatin. Mutations in the Med8-Med18-Med20 submodule did not alter Mediator occupancy at centromeres; however, they led to an increased recruitment of RNA polymerase II to centromeres and reduced levels of centromeric H3K9 methylation accounting for the centromeric desilencing. Further, we observed that Med18 and Med20 were required for efficient processing of *dh* transcripts into siRNA. Consistent with defects in centromeric heterochromatin, cells lacking Med18 or Med20 displayed elevated rates of mitotic chromosome loss.

**Conclusions:**

Our data demonstrate a role for the Med8-Med18-Med20 Mediator submodule in the regulation of non-coding RNA transcription at *Schizosaccharomyces pombe* centromeres. In wild-type cells this submodule limits RNA polymerase II access to the heterochromatic DNA of the centromeres. Additionally, the submodule may act as an assembly platform for the RNAi machinery or regulate the activity of the RNAi pathway. Consequently, Med8-Med18-Med20 is required for silencing of centromeres and proper mitotic chromosome segregation.

## Background

Mediator is a large (approximately 1 MDa) protein complex that conveys regulatory signals to RNA polymerase II (Pol II). The *Saccharomyces cerevisiae* Mediator was the first to be characterized but Mediators have since then been described in many other species. A comparative genomics approach of approximately 70 eukaryotic genomes shows that although its exact subunit composition varies, Mediator is conserved across the eukaryotic kingdom [1]. The *Schizosaccharomyces pombe* Mediator consists of at least 20 subunits, all of which appear to have orthologues in *Drosophila melanogaster, Caenorhabditis elegans* and *Homo sapiens*[2].

Three distinct domains (head, middle and tail) have been identified by electron microscopy on single Mediator particles from *S. cerevisiae*[3]. Electron microscopy on the *S. pombe* Mediator also shows a head and a middle domain, but no tail domain consistent with the lack of *S. pombe* orthologues of the *S. cerevisiae* tail components [4]. The head domain can structurally be further divided (for example, a head domain submodule consisting of Med8-Med18-Med20 is found in both *S. pombe* and *S. cerevisiae*) [5,6]. In *S. pombe,* Med27 may also be part of this submodule [7]. A specific role for the Med8-Med18-Med20 submodule has hitherto not been described, although it is known from work in *S. cerevisiae* that Med18-Med20 interacts directly with the RNA Pol II subunits Rpb4 and Rpb7 [8].

**Figure 1:**
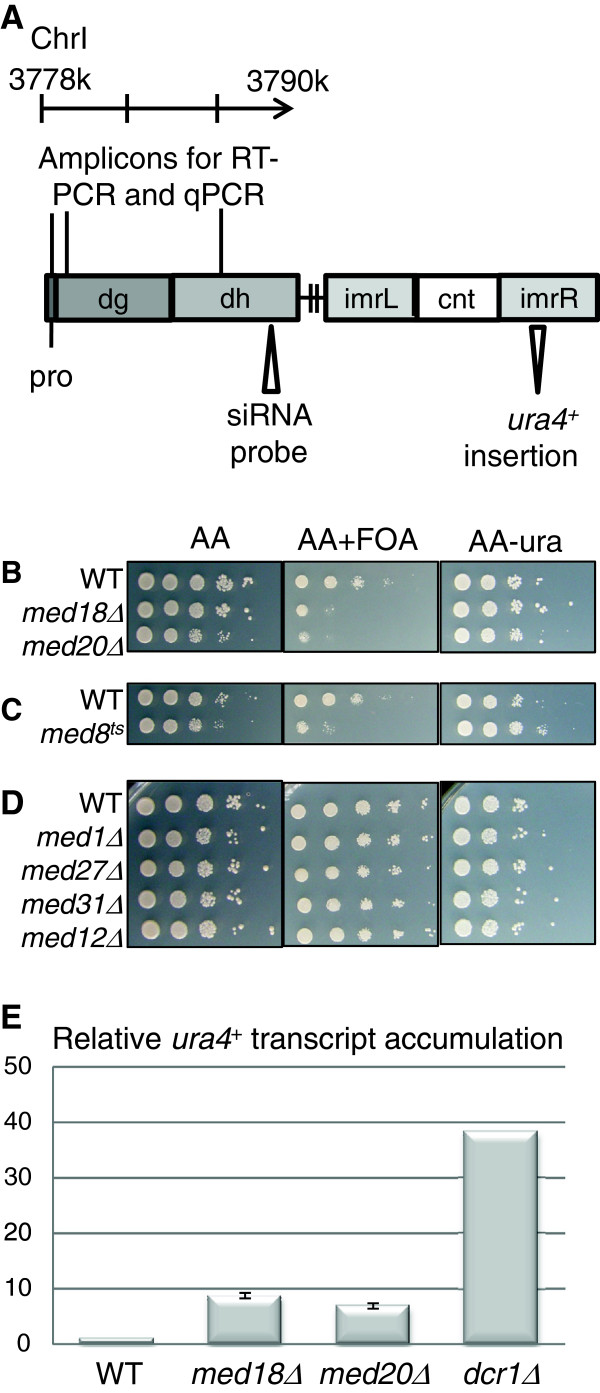
**Centromeric silencing is alleviated by mutations in the Med8-Med18-Med20 submodule.** (**A**) Schematic representation of *S. pombe* centromere 1. The insertion site of the *ura4*^*+*^ reporter used below (*imr1R(NcoI)::ura4*^*+*^), the probe for siRNA detection in Figure 3, and amplicons for the various PCRs performed in this study are shown. One position of the putative *dg* promoter (*pro*) is indicated relative to the outer repeats (*dg* and *dh*) of centromere 1. The crossed line represents an array of *dg* and *dh* repeats next to the innermost repeats (*imr*) and the central core (*cnt*). (**B**-**D**) Ten-fold serial dilutions of cell suspensions were spotted onto the indicated media. Plates were incubated at 33°C for (**B**) and (D) and at 37°C for the *med8*^*ts*^ mutant in (**C**). Expression of *ura4*^*+*^ permits growth in the absence of uracil and causes sensitivity to 5-FOA. Reduced growth on 5-FOA for the *med18*Δ, *med20*Δ and *med8*^*ts*^ mutants indicates derepression of heterochromatic silencing in these three strains. In contrast, deletion of other non-essential Mediator subunits in (**D**) does not alter growth on 5-FOA. (**E**) Quantification of *ura4*^*+*^ transcript by RT-qPCR confirms derepression of *imr1R(NcoI)::ura4*^*+*^ in the *med18Δ* and *med20Δ* mutants. The actin transcript (*act1*^*+*^) was used for normalization. A *dcr1*Δ strain is shown for comparison. The strains for this figure were: WT (FY498), *med18Δ* (MT42), *med20Δ* (MT26), *med8*^*ts*^ (MT31) *med1*Δ (MT13), *med27*Δ (MT11), *med31*Δ (MT14), *med12*Δ (MT6), and *dcr1*Δ (TP480).

Like metazoans, *S. pombe* has large and complex centromeres. *S. pombe* centromeres comprise a central core surrounded by inner and outer repetitive sequences, *imr* and *otr* respectively. The *otr* repeats consist of alternating *dh* and *dg* repeats (Figure 1A). Both *imr* and *otr* are heterochromatic, and reporter genes inserted into the repeats are silenced [9]. Silencing and heterochromatinization of the repeats depend on the RNA interference (RNAi) pathway [10]. RNAi relies on transcription of the centromeric repeats by RNA Pol II [11]. Centromeric transcripts are processed into siRNA by the RNAi machinery, leading to the recruitment and accumulation at centromeres of several interacting protein complexes and histone-modifying enzymes. These include the Argonaute-containing complex RITS [12], the RNA-dependent RNA polymerase complex RDRP [13], the Clr4 histone 3-lysine 9 (H3K9) methyltransferase complex CLRC [14-18] and the trimethyl H3K4 demethylase Lid2 [19]. These protein complexes are capable of interacting with modified nucleosomes and, possibly, non-coding centromeric RNAs and both types of interactions are believed to be required for proper heterochromatin formation and chromosome segregation [20,21].

In spite of the central role played by non-coding RNAs at *S. pombe* centromeres, little is known regarding the regulation of transcription in pericentromeric repeats. Transcription of the *dg* and *dh* repeats peaks during the S-phase of the cell cycle in a window where histone modifications change as a consequence of other cell-cycle regulated events [22-24]. Presently, only one promoter controlling transcription of a centromeric repeat has been described [25]. Consistent with transcription being performed by RNA Pol II, centromeric transcripts are poly-adenylated [26] and specific mutations in RNA Pol II subunits impair heterochromatin formation [25,27,28]. The involvement of RNA Pol II in heterochromatin assembly indicates that the Mediator complex may also play a role in heterochromatin biology. Indeed, deletion of *med1*^+^ or *med6*^+^ was shown to lead to a moderate loss of centromeric silencing in a high throughput study [29]. Further, Med15 was shown to interact with the chromatin-remodelling factor Hrp1 thus associating chromatin state with the Mediator complex [30]. Mediator has also been associated with regulation of chromatin in HeLa cells as Med12, Med19 and Med26 interact with the silencing factor REST and the methyltransferase G9a, which methylates H3K9 at target genes [31,32]. Here, we present a systematic analysis of *S. pombe* Mediator deletion mutants in relation to heterochromatin, and we identify roles played by the Med8-Med18-Med20 submodule in the transcriptional regulation of centromeric repeats and thus in heterochromatin formation, centromere function and chromosome segregation.

## Results and discussion

### A subset of Mediator subunits are required for silencing of a centromeric *ura4*^*+*^ reporter gene

Genes encoding non-essential subunits of Mediator were individually deleted in FY498, a strain with the *S. pombe ura4*^*+*^ gene ectopically inserted in the centromere of chromosome 1, at *imr1R(NcoI)*[33]. In addition, a *med8*^*ts*^ allele [34] was crossed into FY498. We found that silencing of *ura4*^*+*^ at *imr1R(NcoI)* depends on all three components of the Med8-Med18-Med20 Mediator submodule, whereas the other four Mediator subunits tested (Med1, Med12, Med27, and Med31) were dispensable for silencing *ura4*^*+*^ at this location (Figure 1B-D). A variegated phenotype was observed for both *med18*Δ and *med20*Δ as some clones showed a robust silencing of *ura4*^*+*^ whereas others showed only weak silencing. Likewise, deletion of *med1*^+^ did occasionally show derepression of centromeric *ura4*^*+*^; however, this was a modest phenotype compared to the phenotype of *med18Δ* and *med20Δ.* Quantification of *ura4*^*+*^ transcript by RT-qPCR confirmed derepression of *imr1R(NcoI)::ura4*^*+*^ in strains with a compromised Med8-Med18-Med20 submodule (Figure 1E).

### *dh* and *dg* transcripts accumulate in the absence of Med18 or Med20

**Figure 2:**
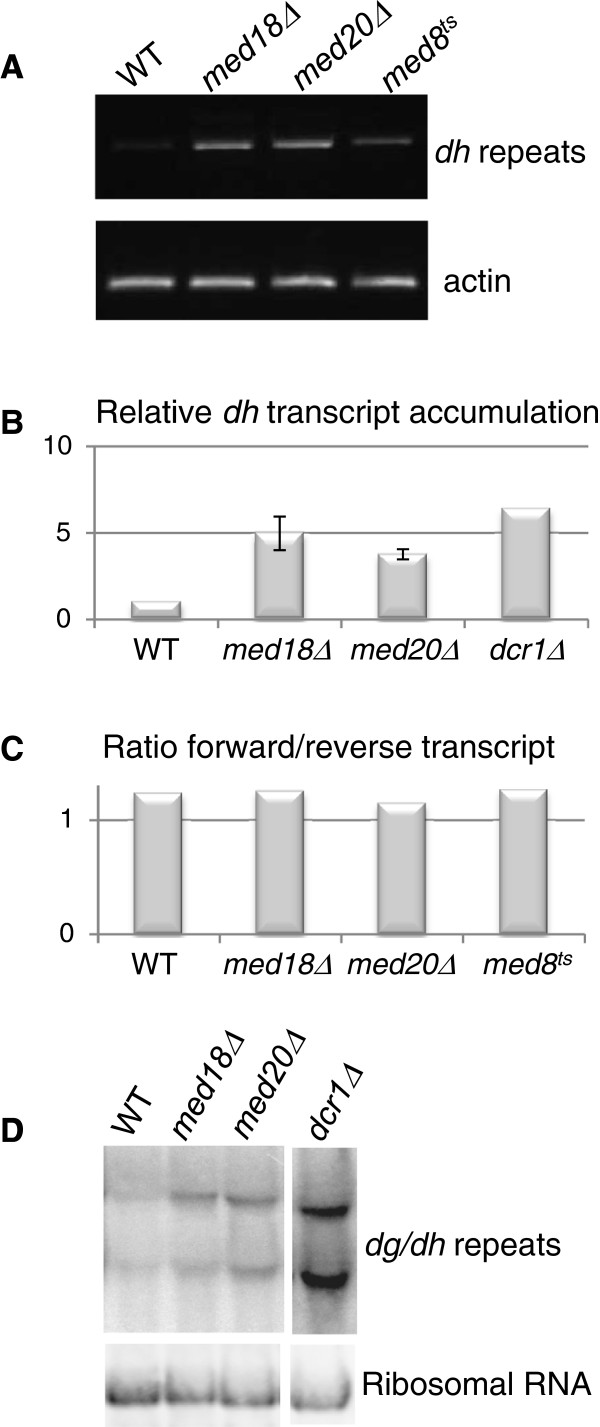
**Mutations in the Med8-Med18-Med20 submodule cause an accumulation of centromeric transcripts.** (**A**) The steady-state level of centromeric non-coding RNA was estimated by RT-PCR in the indicated mutants. The actin transcript was used as reference. (**B**) RT-qPCR shows that the *dh* transcript accumulates in *med18Δ* and *med20Δ* strains. (**C**) Strand specific RT-PCR shows that *med18Δ* and *med20Δ* strains have wild-type ratios of forward to reverse transcripts. (**D**) Northern blot analysis shows that the length of major centromeric transcripts is unchanged in the mutants. The strains for this figure were: WT (FY498), *med18Δ* (MT42), *med20Δ* (MT26), *med8*^*ts*^ (MT31), and *dcr1Δ* (TP480).

To test whether the derepression observed with the *ura4*^*+*^ reporter extends to the native centromeric repeats, RT-PCRs and qPCRs were performed to compare the abundance of centromeric transcripts in the wild type and the *med18* and *med20* deletion strains. We found that *dh* and *dg* transcripts accumulated following deletion of *med20*^*+*^ or *med18*^*+*^ (Figure 2A, B, and data not shown). The changes in transcript levels did not appear to be strand specific (Figure 2C). The size of the transcripts from the *dh* and *dg* repeats estimated by Northern blotting for the *med18Δ* and *med20Δ* mutants were similar to wild type (Figure 2D). Combined, these data indicate that the Med8-Med18-Med20 submodule is not involved in choosing promoters or transcription termination sites but that it more likely influences transcription rate or efficiency of transcript processing.

### The steady-state level of centromeric siRNA depends on Med18 and Med20

**Figure 3:**
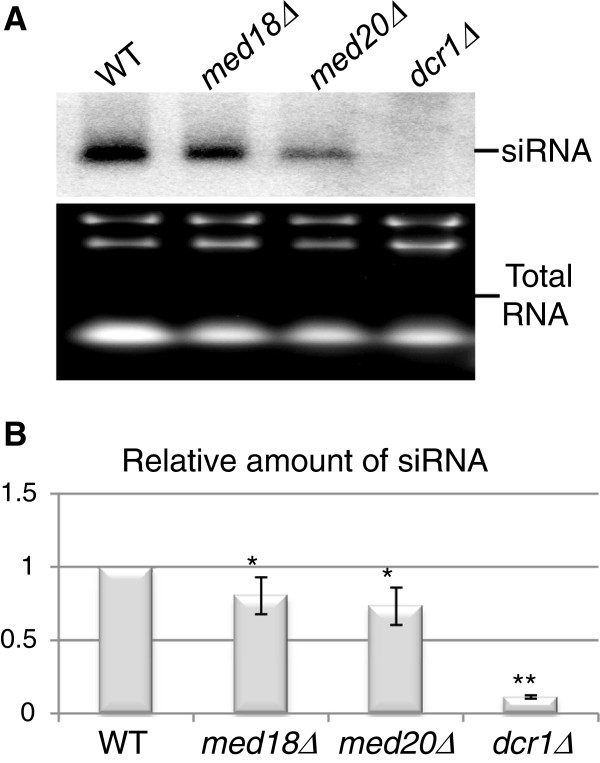
**siRNA levels in *med18***^***+***^**and *med20***^***+***^**deletion strains.** (**A**) Representative Northern blot of siRNA in wild type and indicated mutants. Total RNA was run on a 17.5% polyacrylamide/7M urea gel, blotted and hybridized as described in Materials and Methods. Ethidium-bromide staining of the same RNA preparations was used as loading control. (**B**) Quantification of the blots (n = 4) *P <0.05; **P =5.2e to −12. The strains for this figure were: WT (FY498), *med18Δ* (MT42), *med20Δ* (MT26), and *dcr1Δ* (TP480).

The increased abundance of *dh* and *dg* transcripts in *med18Δ* and *med20Δ* mutants could be explained by either elevated transcription or reduced processing of the transcripts. To estimate whether *dh* transcripts were processed into siRNA, we performed Northern blot analyses on total RNA. A random-primed probe was generated from a PCR fragment corresponding to a region of the *dh* repeats known to yield high levels of siRNA [35]. Using this probe clearly showed that the processing of centromeric transcripts was not abolished when *med18*^*+*^ or *med20*^*+*^ was deleted as siRNA remained easily detectable in the mutants. However, the deletion strains contained approximately 20 to 30% less siRNA than the wild-type control indicative of a partial impairment of siRNA biogenesis in the two mutants. A strain without *dcr1*^*+*^ did not show any detectable siRNA in this assay (Figure 3A, B). Thus, the increase in non-coding RNA levels did not result in higher, but lower siRNA production indicating that wild-type regulation of *dh* transcription is required for effective *dh* siRNA formation.

### Lack of Med18 or Med20 does not influence Mediator recruitment to centromeres

**Figure 4:**
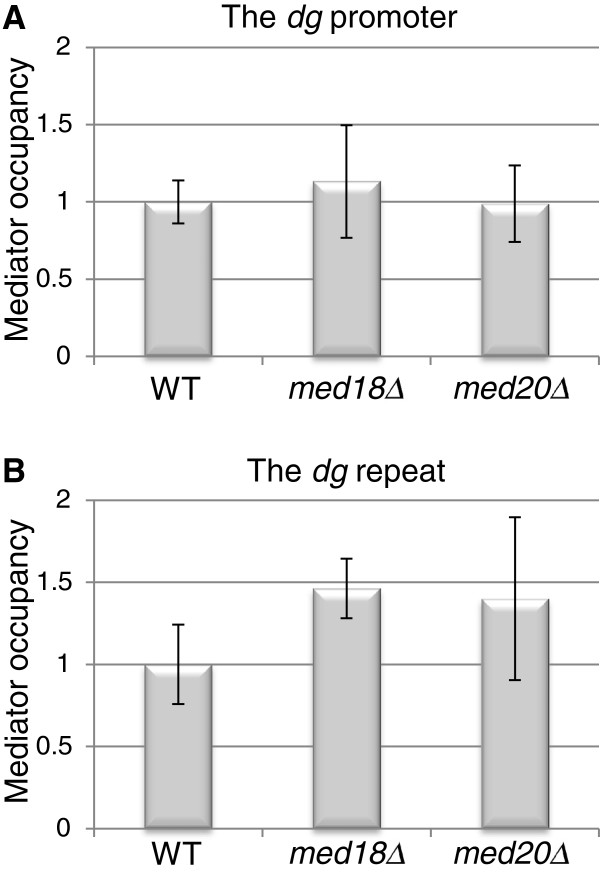
**Mediator occupancy (Med7-TAP) at the centromeric *dg *promoter and *dg *repeat in *med18***^***+***^**and *med20***^***+***^**deletion strains.** ChIP analyses show that the relative Mediator occupancy at (**A**) the centromeric promoter as well as at (**B**) the *dg* repeat is unchanged in *med18Δ* and *med20Δ* mutant strains. The strains for this figure were: WT (FY498), *med18Δ* (MT42), and *med20Δ* (MT26).

The modest decrease in siRNA levels observed in the *med18Δ* and *med20Δ* mutants suggested that reduced processing of centromeric transcripts might not on its own account for the elevated levels of *dh* and *dg* transcripts in these mutants. Elevated transcript levels could also be a consequence of the Med18-Med20-Med8 submodule functioning as a negative regulator of transcription from the *dh* and *dg* repeats in wild-type cells. A single pericentromeric promoter driving expression of *dg* and *dh* repeats has been described in the literature [25]. We estimated Mediator occupancy at this promoter and at the *dg* repeat regulated by the promoter by chromatin immunoprecipitation (ChIP). The Mediator subunit Med7 was pulled down followed by qPCRs for promoter and *dg* sequences, respectively. The assay showed that Mediator is associated with the centromeric regions tested and that its association is not affected by deletion of *med18*^+^ or *med20*^+^ (Figure 4). These observations are consistent with a direct role of Mediator at centromeres and suggest that the Med8-Med18-Med20 submodule negatively regulates transcription downstream of Mediator association with centromeres.

### Strains lacking Med18 or Med20 display increased RNA Pol II occupancy on the dg promoter and on the dg repeat itself

**Figure 5:**
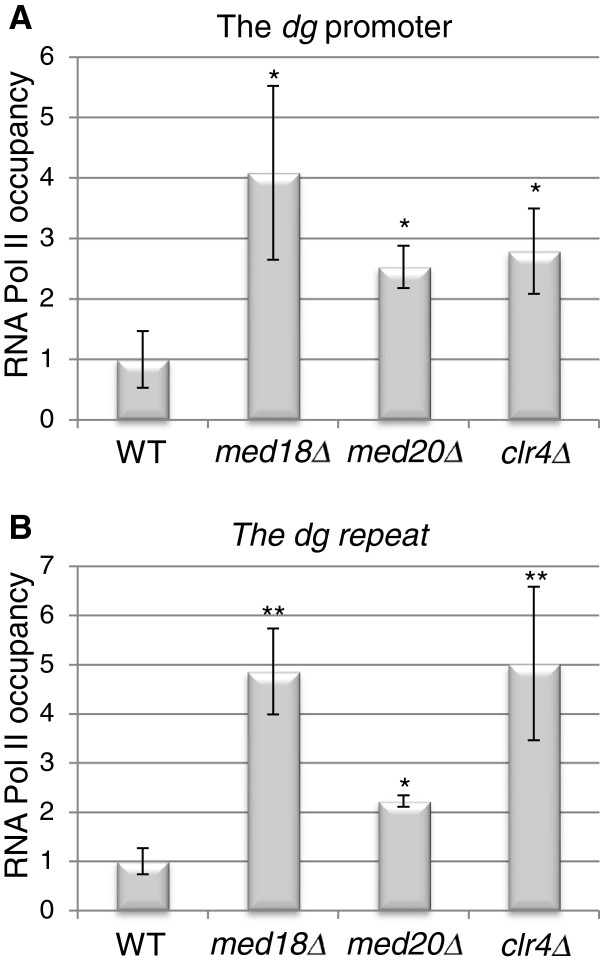
**RNA Pol II occupancy at the centromeric promoter and *dg *repeat in *med18***^***+***^**and *med20***^***+***^**deletion strains.** ChIP analyses show that compared to wild-type, the RNA Pol II occupancy at (**A**) the *dg* centromeric promoter as well as at (**B**) the *dg* repeat is increased in *med18*^*+*^ and *med20*^*+*^ deletion strains. For comparison, the RNA Pol II occupancy in a *clr4Δ* deletion strain is also shown. *P <0.004; **P <1e to −6. The strains for this figure were: WT (FY498), *med18Δ* (MT42), and *med20Δ* (MT26), and *clr4Δ* (PG3423).

One well-documented function of the Mediator complex is to regulate RNA Pol II activity [36]. We therefore assayed RNA Pol II occupancy in pericentromeric repeats by ChIP in wild-type, *med18Δ, med20Δ*, and *clr4Δ* cells. ChIP-qPCR performed both on the putative *dg* promoter and on the *dg* repeat showed an RNA Pol II enrichment of two and five fold in *med20*Δ and *med18*Δ, respectively, compared to wild-type. The enrichment of RNA Pol II in *med18*Δ is similar to the enrichment seen in a *clr4Δ* strain in a parallel experiment (Figure 5). The fact that Clr4 limits RNA Pol II occupancy at centromeres was previously reported [24] but the precise mechanism through which exclusion occurs is unknown. Our results strengthen the view that the Med8-Med18-Med20 submodule negatively regulates non-coding RNA transcription at centromeres by reducing the ability of Mediator to recruit RNA Pol II. This process might be part of the mechanism through which the Clr4 H3K9 methyltransferase excludes RNA Pol II from centromeres.

### Desilencing of centromeric heterochromatin in *med18* and *med20* mutants correlates with decreased H3K9 methylation

**Figure 6:**
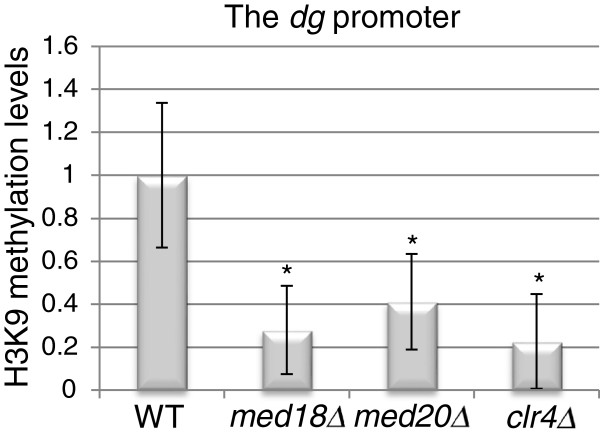
**Mutations in the Med8-Med18-Med20 submodule compromise H3K9 methylation at the centromeric *dg *promoter.** ChIP analyses show that the level of H3K9 dimethylation at the centromeric *dg* promoter is reduced in *med18*Δ and *med20Δ* mutants relative to wild-type. A *clr4Δ* strain was processed in parallel for comparison. *P <0.003. The strains for this figure were: WT (FY498), *med18*Δ (MT42), *med20*Δ (MT26), and *clr4Δ* (PG3423).

The increased abundance of non-coding centromeric transcripts in strains deleted for *med18*^*+*^ or *med20*^*+*^ prompted us to investigate the methylation levels of histone H3K9. Figure 6 shows that dimethylation of H3K9 was reduced on the putative *dg* promoter in the *med18Δ* and *med20Δ* mutants. H3K9 methylation at the *dg* repeat next to the promoter was also reduced, but less significantly (data not shown). This observation is consistent with the Med8-Med18-Med20 submodule acting upstream of Clr4 to facilitate H3K9 methylation. The Med8-Med18-Med20 submodule might recruit Clr4, which would in turn inhibit RNA Pol II through H3K9 methylation. Because RNAi-directed heterochromatin formation forms a self-enforcing loop, indirect effects could also account for reduced H3K9me in Mediator mutants as depicted in the model we present in a later section.

### Mutations in the Med8-Med18-Med20 submodule and deletion of *clr4*^*+*^ lead to similar changes in transcription profile

More generally, we noticed that the genome-wide expression profiles of *clr4* and Mediator mutants display striking similarities indicating the Med8-Med18-Med20 submodule and H3K9me act in concert at many locations other than centromeres. A total of 42/110 genes upregulated more than 1.5x in *clr4-481*[26] are upregulated more than 2x in the *med8*^ts^ mutant ([37]; 164 genes are upregulated more than 2x in the *med8*^ts^ mutant). A total of 24/58 genes upregulated more than 1.5x in *clr4Δ* are upregulated more than 2x in the *med8*^ts^ mutant. These genes are enriched in large subtelomeric regions extending approximately 100 kb into chromosomes 1 and 2; 39/164 genes upregulated more than 2x in the *med8*^ts^ mutant are subtelomeric. These regions share properties with centromeric heterochromatin [26,38,39] The same subtelomeric gene clusters are controlled by Spt6 [40] suggesting Spt6, Clr4, and the Med8-Med18-Med20 Mediator submodule cooperate in heterochromatic gene silencing both at centromeres and at other chromosomal locations.

### Chromosome segregation is affected in *med18*Δ **and***med20*Δ **strains**

**Figure 7:**
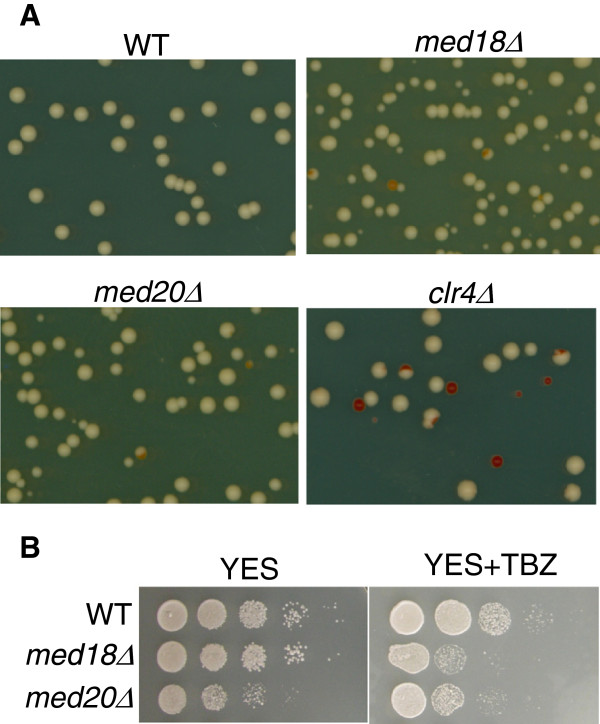
**Deletion of *med18***^***+***^**or *med20***^***+***^**impairs centromere function.** (**A**) A non-essential mini-chromosome, Ch16m23::*ura4*^+^-Tel[72]*,* is frequently lost in strains deleted for *med18*^*+*^, *med20*^*+*^ or *clr4*^*+*^. Cells containing the mini-chromosome form white colonies on medium with low concentration of adenine while cells lacking the mini-chromosome form red colonies. Loss of the mini-chromosome in the first cell division after plating results in a half-sectored colony. (**B**) Deletion of *med18*^*+*^ or *med20*^*+*^ renders the cells sensitive to the microtubule destabilizing agent thiobendazole (12 μg/ml). The strains for this figure were: WT (FY520), *med18*Δ (TP527), *med20*Δ (TP527), and *clr4Δ* (PG3420).

Defects in heterochromatin impair the association of cohesins with centromeric regions and increase mitotic and meiotic chromosome loss [33,41,42]. To further investigate whether mutations in the Mediator complex affect the functionality of centromeres, we measured the rate of mitotic loss of a non-essential mini-chromosome, Ch16m23::*ura4*^+^-Tel[72] [43], in *med18Δ*, *med20Δ* and wild-type strains. For comparison we included a *clr4Δ* strain in the analysis. Chromosome segregation was affected in *med18Δ* and *med20Δ* mutants corroborating the alleviated-silencing phenotype of these mutants. These strains lost their mini-chromosome in approximately 0.3 to 0.8% of cell divisions compared to approximately 4% in a *clr4*Δ background and more than 0.025% in wild-type cells (Figure 6A and Table 1). These changes correspond to a 32- and 12-fold increase in mini-chromosome loss rates in *med18Δ* and *med20Δ*, respectively, compared to wild-type. In addition, strains without Med18 or Med20 were sensitive to the microtubule destabilizing agent thiobendazole (Figure 7B), further implicating Med18 and Med20 as crucial factors for maintaining centromere function.

**Table 1:** **Mini-chromosome loss rate is higher in strains deleted for *med18***^***+ ***^**or *med20***^***+***^

**Strain**	**Half sectored**	**White**	**Loss Rate**
WT	1	4012	0,025%
*clr4Δ*	85	2181	3,9%
*med18Δ*	26	3195	0,8%
*med20Δ*	7	2339	0,3%

## Conclusions

**Figure 8:**
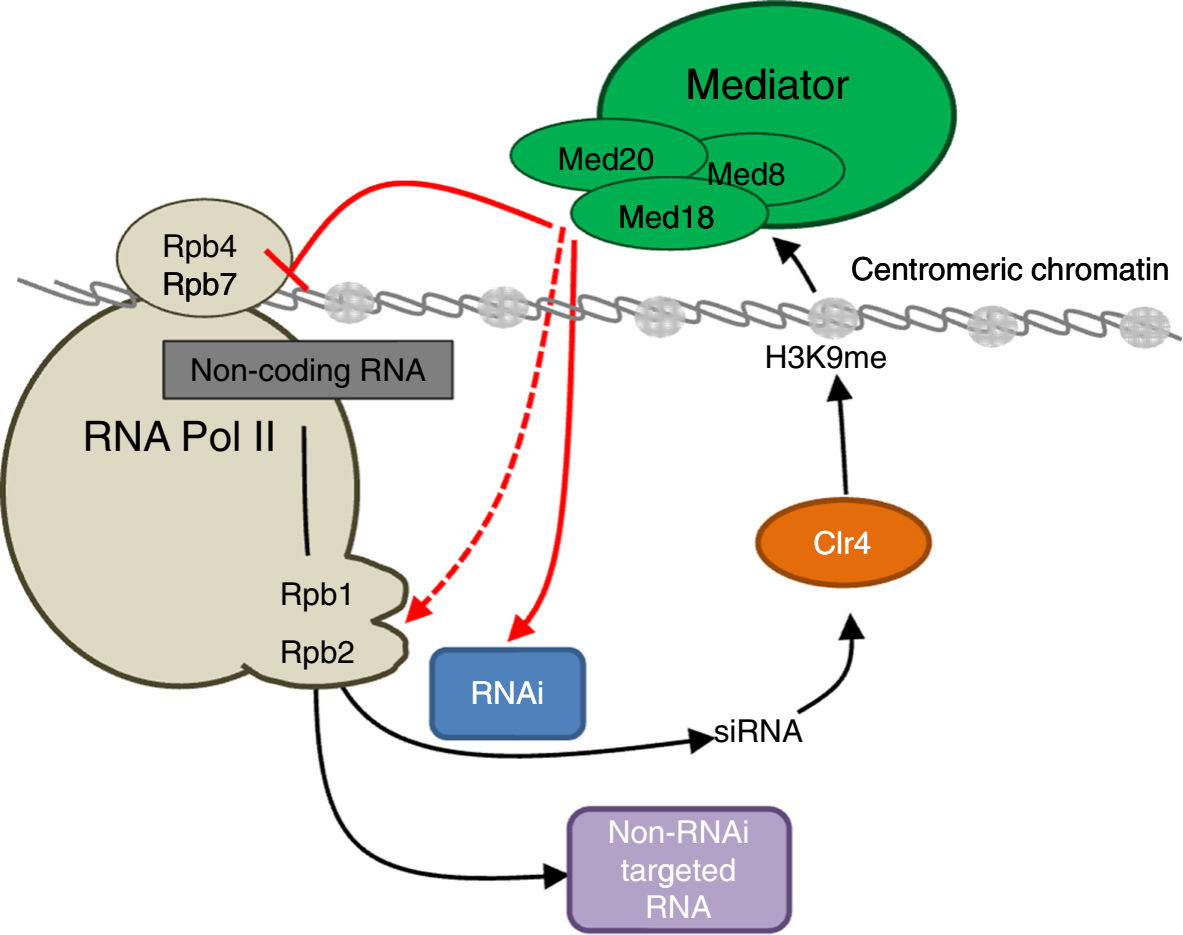
**Model illustrating the effect of the Med8-Med18-Med20 submodule on heterochromatin.** Med8-Med18-Med20 may block recruitment of RNA Pol II to the centromeric chromatin by interacting with Rpb4/Rpb7. Additionally, the submodule may stimulate the activity of RNAi and thus influence the methylation level of H3K9 in centromeric chromatin. Further, Med8-Med18-Med20 in concert with Rpb1/Rpb2 may decide the fate of non-coding transcripts by directing them towards the RNAi machinery or to other downstream processes. See text for details.

The central observations presented here, that long centromeric non-coding RNAs accumulate in mutants compromised in the Med8-Med18-Med20 submodule of Mediator, that centromeric H3K9me is reduced in these mutants, and that the levels of siRNAs are not dramatically altered but, if anything, slightly reduced in the mutants can be understood as depicted in Figure 8. The model in Figure 8 proposes that one role of the Med8-Med18-Med20 Mediator submodule is to prevent the recruitment of RNA Pol II to centromeric heterochromatin. By analogy with *S. cerevisiae* where the Med8-Med18-Med20 submodule was reported to interact with the Rpb4/Rpb7 RNA polymerase II subunit complex [8], we propose that *S. pombe* Med8-Med18-Med20 also interacts with Rpb4/Rbp7. The structural studies monitoring Med18-Med20 interaction with Rpb4/Rpb7 in *S. cerevisiae* reveal that Med18-Med20 modulates the conformation of RNA Pol II, regulating its interaction with DNA. Thus, a mutation in the Med8-Med18-Med20 submodule is likely to affect RNA Pol II function. In *S. pombe*, the Rpb7 subunit of RNA Pol II is required for initiation of transcription of centromeric non-coding RNAs. In the *rpb7-G150D* mutant reduced transcription initiation at centromeres leads to compromised heterochromatin which allows for more spurious transcription and accumulation of non-coding transcripts [25]. We propose that the Med8-Med18-Med20 submodule limits centromeric transcription in wild-type cells by inhibiting transcription initiation through Rbp4/Rpb7.

Mechanistically, the interaction between the Med18-Med20 sub-complex and the Rpb4/Rpb7 sub-complex of Pol II has been proposed to alter the conformation of the Pol II clamp domain to facilitate opening of its active-site cleft and thereby the access of promoter DNA to the Pol II cleft [44]. This interaction would facilitate pre-initiation-complex (PIC) formation. We suggest that in heterochromatin specific interactions of other components with Mediator and/or Pol II might prevent clamp movement and thereby the productive interaction of Pol II with DNA.

Since the above proposed function of Med8-Med18-Med20 might not account for the decrease in siRNA or H3K9me in the mutants, we suggest that the Med8-Med18-Med20 submodule also facilitates the processing of long non-coding RNAs into siRNA. This second function might be carried out together with the two largest *S. pombe* RNA Pol II subunits, Rpb1 [28] and Rpb2 [27]. A mutation in Rbp2, *rpb2-m203,* increases the steady-state levels of centromeric transcripts and reduces siRNA to undetectable levels [27]. The *rpb2-m203* phenotype has been taken to suggest that Rpb2 provides an interaction interface with RNAi complexes and/or a means of distinguishing non-coding centromeric transcripts from mRNA, triggering processing of the former into siRNA [27]. This presumed function of RNA Pol II, which would be compromised by the *rpb2-m203* mutation, may also be affected by mutation in the Med8-Med18-Med20 submodule. A non-mutually exclusive possibility is that Med8-Med18-Med20 facilitates processing of centromeric non-coding RNA into siRNA together with Rpb1 [28]. The *S. pombe* C-terminal domain of Rpb1 contains 28 conserved YSPTSPS repeats acting as an assembly platform for various mRNA processing factors, thus coupling transcription to pre-mRNA processing and export. A mutant form of Rpb1 (*rpb1-11*) retaining 16 of the 28 hepta-repeats apparently does not affect transcription of the pericentromeric repeats, but nevertheless compromises downstream RNAi function [28]. As for Rpb2, given the ubiquitous interactions between the Mediator complex and active RNA Pol II, it seems plausible that a mutation in Med8-Med18-Med20 might disturb the Rpb1-dependent RNAi machinery assembly function. Alternatively, the Med8-Med18-Med20 submodule might itself be a site where pre-siRNA processing is regulated.

Consistent with our conclusions, a very recent study by Zhu and colleagues [45], published during the writing of this article, reports an accumulation of centromeric non-coding RNA and reduced processing of the *dh* repeat transcript into siRNA in a *med20Δ* strain. In addition, an independent large-scale epistasis map revealed genetic interactions between subunits of the Mediator and RNAi and heterochromatin components [29]. Neither *med8* nor *med18* mutants were included in this screen but probing the bioGRID [46] with Osprey [47] lists 101 genetic interactions for *med20* including interactions with *dcr1*^*+*^*, ago1*^*+*^*, hrr1*^*+*^*, swi6*^*+*^*, cid12*^*+*^, *clr3*^*+*^*, hda1*^*+*^*, hst2*^*+*^*, pob3*^*+*^, *set3*^*+*^*, swc2*^*+*^ and *epe1*^*+*^. These interactions with heterochromatin-associated factors are fully consistent with the notion that the Med8-Med18-Med20 submodule participates in *S. pombe* heterochromatin formation. The data presented here, which are corroborated by Carlsten *et al*. [45], clearly demonstrate a role for Mediator in regulating centromeric chromatin.

## Methods

### Strains and primers

The *S. pombe* strains used in this study are listed in Table 2 and the primers are listed in Table 3.

**Table 2:** ***Schizosaccharomyces pombe s*trains used in the study**

**Name**	**Genotype**	**Source**
FY498	*h*^*+*^*ura4-DS/E ade6-210 imr1R(NcoI)::ura4*^*+*^*ori1*	[9]
MT6	*h*^*+*^*ura4-DS/E ade6-210 imr1R(NcoI)::ura4*^*+*^*ori1 med12Δ::KanMX*	This study
MT11	*h*^*+*^*ura4-DS/E ade6-210 imr1R(NcoI)::ura4*^*+*^*ori1 med27Δ::KanMX*	This study
MT13	*h*^*+*^*ura4-DS/E ade6-210 imr1R(NcoI*)::*ura4*^+^*ori1 med1Δ*::*KanMX*	This study
MT14	*h*^+^*ura4*-*DS*/*E ade6*-*210 imr1R*(*NcoI*)::*ura4*^+^*ori1 med31Δ*::*KanMX*	This study
MT26	*h*^+^*ura4*-*DS*/*E ade6*-*210 imr1R*(*NcoI*)::*ura4*^+^*ori1 med20Δ*::*KanMX*	This study
MT42	*h*^+^*ura4*-*DS*/*E ade6*-*210 imr1R*(*NcoI*)::*ura4*^+^*ori1 med18Δ*::*KanMX*	This study
TP480	*h*^+^*ura4*-*DS*/*E ade6*-*210 imr1R*(*NcoI*)::*ura4*^+^*ori1 dcr1Δ*::*KanMX*	This study
FY520,	*h*^+^*ura4*-*DS*/*E ade6*-*210*/*216* Ch16m23::*ura4*^+^-Tel[72]	[43]
TP528	*h*^+^*ura4*-*DS*/*E ade6*-*210*/*216* Ch16m23::*ura4*^+^-Tel[72] *med20Δ*::*KanMX*	This study
TP527	*h*^+^*ura4*-*DS*/*E ade6*-*210*/*216* Ch16m23::*ura4*^+^-Tel[72] *med18Δ*::*KanMX*	This study
PG3420	*h*^*A*^ Ch16m23::*ura4*^+^-Tel[72] *leu1*-*32 ura4*-*DS*/*E ade6*-*210*/*216 clr4Δ*::*LEU2*	[17]
PG3423	*mat1*-*Msmt*-*0 mat2*-*P*(*XbaI*)::*ura4*^+^*leu1*-*32 ura4*-*DS*/*E ade6*-*210 clr4Δ*::*LEU2*	[17]

**Table 3:** Oligonucleotides used in the study

**Name**	**Sequence**
dhH-siRNA	TACTGTCATTAGGATTAGCACA
Cen-dh-FOR2	CGACAAACTTCATGTTACAAGTC
GTO265	GCTATTCAGCTAGAGCTGAGGG
GTO266	CTTCGACAACAGGATTACGACC
GTO223	GAAAACACATCGTTGTCTTCAGAG
GTO226	TCGTCTTGTAGCTGCATGTGA
OKR70	GGCATCACACTTTCTACAACG
OKR71	GAGTCCAAGACGATACCAGTG
Act1 q-PCR FW	CTGTTTTGTCTTTGTATGCC
Act1 q-PCR RV	TAAGGTAGTCAGTCAAGTCA
dhA q-PCR FW	GCAAACAGACCCTCATACAG
dhA q-PCR RV	CAAGGACTAAGCCCAAGCAC
ura4 q-PCR FW	CGTGGTCTCTTGCT TTGG
ura4 q-PCR RV	GTAGTCGCTTTGAAGGTTAGG
p33F	TGCAAGTGGAAAGTGGCTTCA
p33R	TCGACCACCCTGACTTGTTCTC
p30F	CCTGTTGATTCGGCACCTTTG
p30R	TGGAGAACGACTGTGAAGAGA
oMiT127	CCGAAAGCCTCGATATCATC
oMiT128	GAGCATGGTGGTGGTTATGG
oMiT142	ACCGTAGTACGACGATGATGTGTTT
oMiT143	ACATTCCGCACAAGGTCTAGTACA

### RT-PCR/qPCR

RNA extraction and RT-PCR were as in [48] except for the final step where quantification was performed by ethidium-bromide staining using a Bio-Rad Laboratories imaging station and the Quantity One image analysis software (Bio-Rad Laboratories, Hercules, CA, USA). Primer sequences are listed in Table 3. For RT-PCR, the oligonucleotides GTO-265 and GTO-266 were used to amplify *ura4*^+^ and *ura4-DS/E*; GTO-223 and GTO-226 were used to amplify RNA originating from centromeric repeats or mating-type region; OKR70 and OKR71 were used to amplify actin mRNA. Strand-specific RT-PCR was achieved by using GTO-226 to prime reverse transcription on centromeric forward transcripts or GTO-223 on centromeric reverse transcripts prior PCR amplification.

RNA used in RT-qPCR was isolated using an RNeasy™ mini kit (Qiagen, Hilden, Germany) and an RNase-Free DNase set (Qiagen, Hilden, Germany). Reverse transcription of the purified RNA was performed using the RevertAid^TM^ First Strand cDNA Synthesis Kit (Thermo Fisher Scientific, Waltham, MA, USA) and random hexamer primers. qPCR was performed on a CFX96 real time PCR system (Bio-Rad Laboratories, Hercules, CA, USA) using the QuantiTect SYBR Green PCR kit (Qiagen, Hilden, Germany) supplied with SYBR Green Reference Dye. Three technical replicates were performed for each of the biological triplicates. Technical replicates with standard deviations above 10% were repeated or excluded from the experiment. Primers used to amplify *act1*^*+*^ and the *dh* repeat are shown in Table 3.

### Chromosome-loss assay

Mitotic chromosome loss was assayed as previously described [9] using cells containing the *ade6-M210* allele on chromosome 3 and the *ade6-M216* allele on the nonessential minichromosome Ch16m23::*ura4*^+^-Tel[72] [43]. Cells with this genotype are phenotypically Ade^+^ due to the interallelic complementation between *ade6-M210* and *ade6-M216*. They form white colonies on media containing low concentrations of adenine. Loss of Ch16m23::*ura4*^+^-Tel[72] results in red colonies or sectors. White and sectored colonies were counted following plating of the strains of interest on yeast extract plates to which no adenine had been added. The rate of minichromosome loss was determined as the number of colonies with a red sector equal to or greater than half the colony size (that is, the number of cells having lost their minichromosome at the first division after plating) divided by the number of white or sectored colonies.

### Northern blot

For siRNA Northern blots, total RNA was isolated with Tri Reagent (Sigma Chemical Co., St. Louis, MO, USA) and 20 μg RNA was run on a 17.5% polyacrylamide/7 M urea gel and blotted onto a positive nylon membrane (Roche Diagnostics, Mannheim, Germany). siRNA were detected using a random-primed probe radioactively labeled with [α-^32^P]-dCTP (3000 Ci/mmol, PerkinElmer, Waltham, MA, USA). The template for random priming was a *dh* repeat PCR product amplified from genomic DNA with the dhH-siRNA and Cen-dh-FOR2 primers. Northern blots detecting the *dg* and *dh* repeats were obtained following electrophoresis of 10 μg total RNA prepared by a hot phenol protocol from the strains of interest. The gels used were 1% agarose in MOPS buffer with 6.7% formaldehyde. RNA was blotted onto a Hybond-XL membrane (GE Healthcare, Little Chalfont, United Kingdom). The *dg* and *dh* repeats were detected by a random-primed [α-^32^P]-dCTP radioactively labeled probe made on PCR products amplified from genomic DNA using p30F and p30R (*dh* repeat) or p33F+p33R (*dg* repeat). Hybridizations were performed overnight at 42°C in PerfectHyb PLUS hybridization buffer (Sigma Chemical Co., St. Louis, MO, USA).

### Chromatin immunoprecipitations

ChIP was performed according to standard procedures. Antibodies used to immunoprecipitate RNA Pol II and H3K9me2 were ChIPAb RNA Pol II (Merck Millipore, Billerica, MA, USA) and histone H3 (dimethyl K9) antibody ChIP Grade ab1220 (Abcam, Cambridge, MA, USA), respectively. Protein G Dynabeads were used to pull down the antibody captured proteins. Rabbit Anti-Mouse Immunoglobulins (Dako, Glostrup, Denmark) were covalently coupled to the surface of Dynabeads with the Dynabeads Antibody Coupling Kit (Invitrogen, Life Technologies, Carlsbad, CA, USA) and these beads were used to pull down the Mediator complex through a TAP-tagged Med7. Presence of RNA Pol II, Mediator or dimethyl H3K9 was detected by qPCR using the primers dhA q-PCR FW and dhA q-PCR RV for the *dh* repeat, oMiT142 and oMiT143 for the *dg* repeat, or oMiT127 and oMiT128 for the putative promoter.

## Abbreviations

ChIP: Chromatin immunoprecipitation; RNA Pol II: RNA polymerase II; RT-PCR: Reverse transcription PCR; RT-qPCR: Quantitative reverse transcription PCR.

## Competing interests

The authors declare that they have no competing interests.

## Authors’ contributions

MT, HH and MV carried out the research. MT, GT and SH wrote the manuscript. GT and SH provided guidance in experimental design and interpretation of data. All authors read and approved the final manuscript.
